# Supplementary material for: Nano-Liquid Chromatography with a New Monolithic Column for the Analysis of Coenzyme Q10 in Pistachio Samples
Source: Molecules. 2023 Feb 2;28(3):1423. doi: 10.3390/molecules28031423 (PMC9920066; doi:10.3390/molecules28031423)

Supplementary Information

Nano-Liquid Chromatography with a New Monolithic Column for the Analysis of Coenzyme Q10 in Pistachio Samples

Cemil Aydoğan [1,2,3,*], Büşra Beltekin [1], Nurullah Demir [1], Bayram Yurt [1,3] and Ziad El Rassi [4]

[1] Food Analysis and Research Laboratory, Bingöl University, Bingöl 12000, Türkiye
[2] Department of Chemistry, Bingöl University, Bingöl 12000, Türkiye
[3] Department of Food Engineering, Bingöl University, Bingöl 12000, Türkiye
[4] Department of Chemistry, Oklahoma State University, Stillwater, OK 74078, USA
* Correspondence: cemilaydogan29@gmail.com; Tel.: +90-426-216-19-58; Fax: +90-426-216-00-33

Table of Contents

Figure S1. The sample preparation with ultrasound sonicator BANDELIN homogenizator.

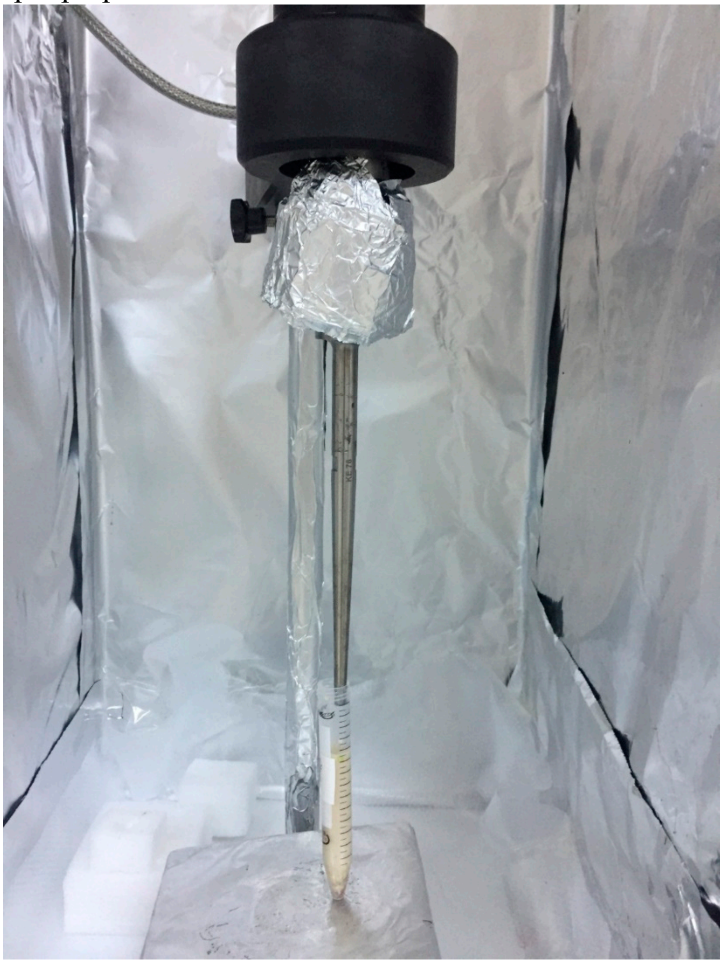

Figure S2. Six pistachio samples to be obtained using the ultrasound sonicator

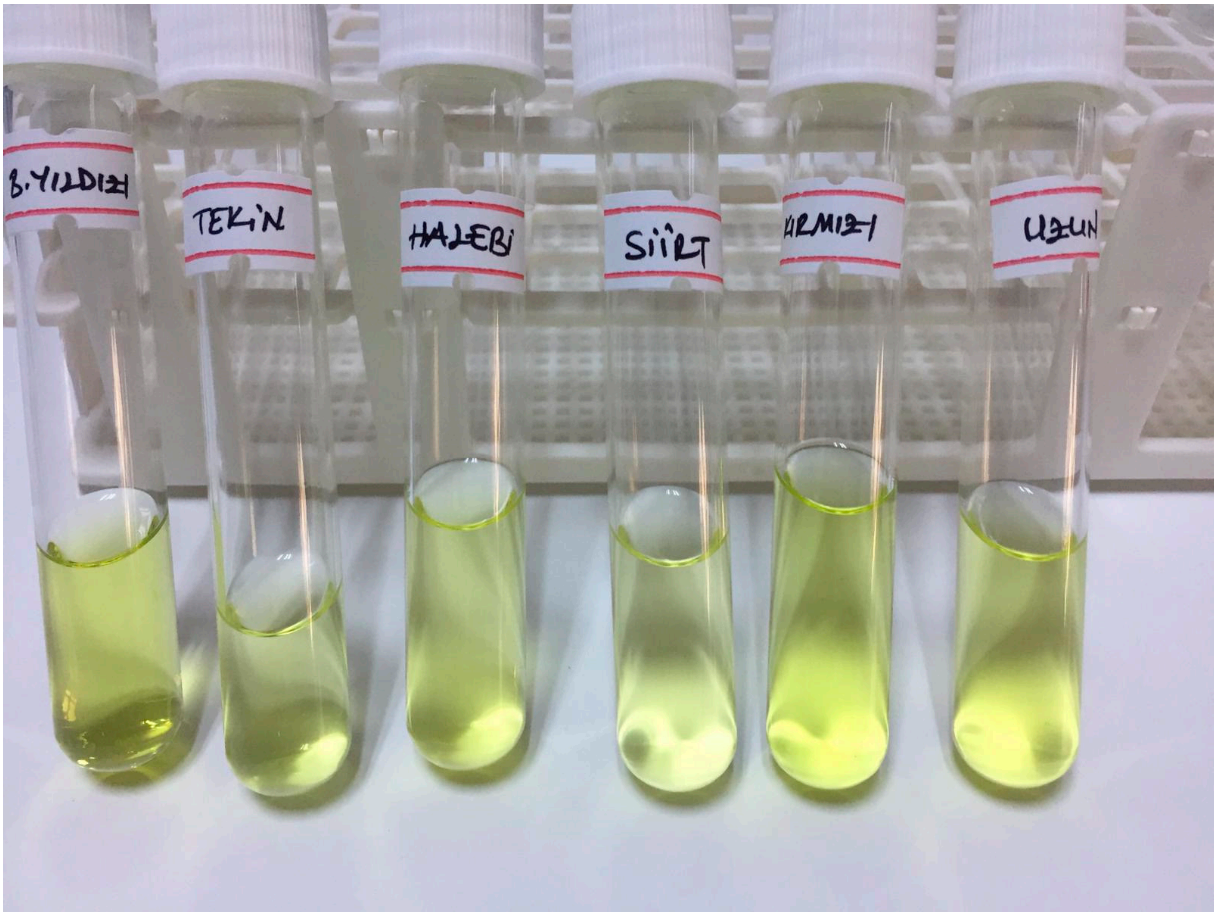

Figure S3. The six different pistachio samples used for CoQ10 determination using developed nano-LC method.

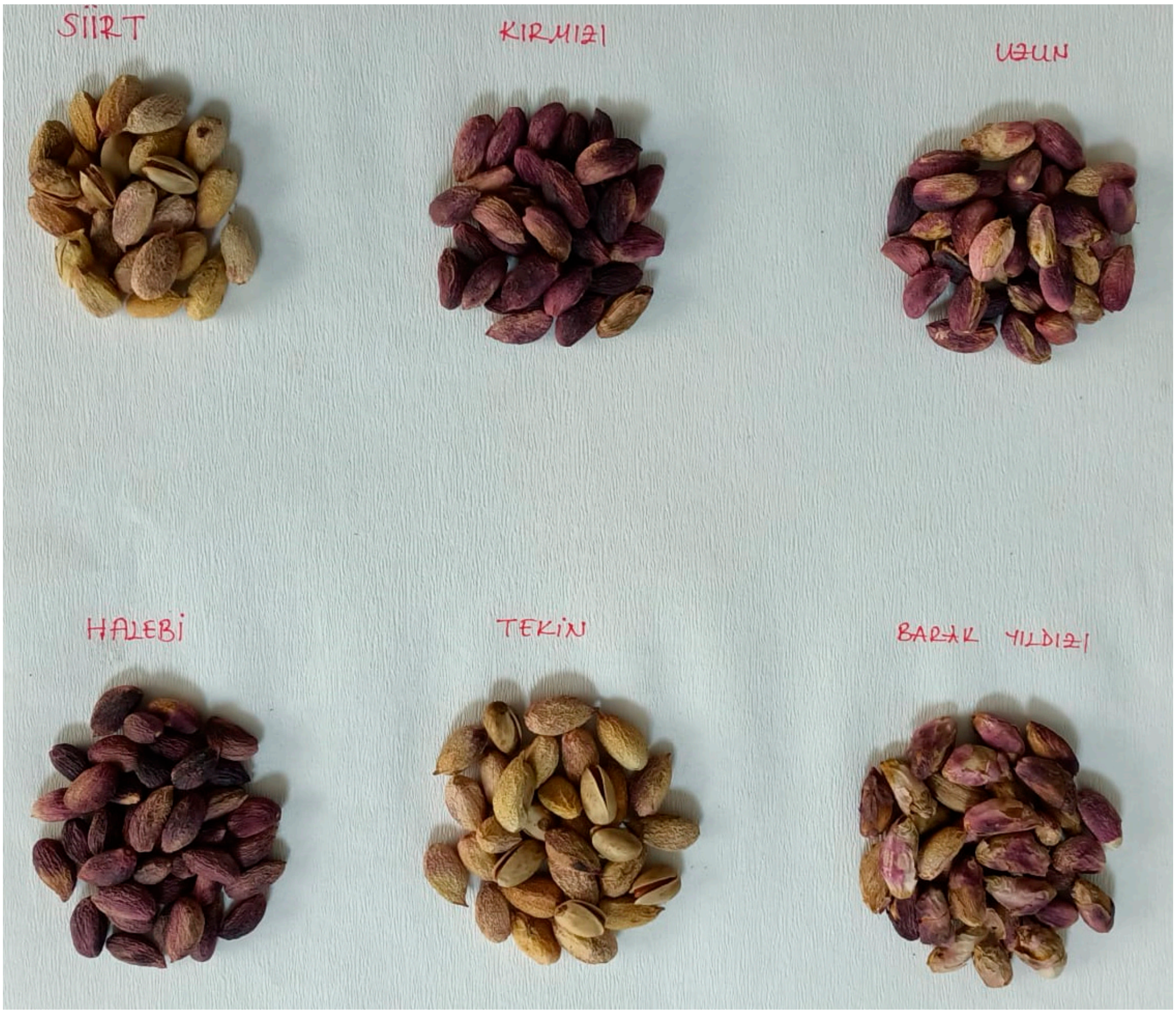

Supplement: Supplementary file 1 [file molecules-28-01423-s001.zip › molecules-2160484-supplementary.pdf]
